# A single nucleotide polymorphism collapses phase variation and drives short-sighted evolution of O-antigen in *Salmonella*

**DOI:** 10.1080/19490976.2026.2721178

**Published:** 2026-08-24

**Authors:** Yiluo Cheng, Wenting Zhang, Rhea Nickerson, Qin Lu, Yunqing Guo, Qiao Hu, Rongrong Zhang, Guoyuan Wen, Qi Huang, Rui Zhou, Huabin Shao, Zhenyu Cheng, Qingping Luo, Tengfei Zhang

**Affiliations:** a Key Laboratory of Prevention and Control Agents for Animal Bacteriosis, Ministry of Agriculture and Rural Affairs, Key Laboratory of Animal Pathogenic Microbiology of Hubei Province, Institute of Animal Sciences and Veterinary Medicine, Hubei Academy of Agricultural Sciences, Wuhan, China; b Department of Microbiology and Immunology, Dalhousie University, Halifax, NS, Canada; c Hubei Hongshan Laboratory, Wuhan, China; d National Key Laboratory of Agricultural Microbiology, College of Veterinary Medicine, Huazhong Agricultural University, Wuhan, China

**Keywords:** Immune escape, O-antigen, phase variation, *Salmonella*, short-sighted evolution

## Abstract

The O-antigen of *Salmonella* is a key virulence determinant and serotype-defining structure, yet the regulatory mechanisms underlying its dynamic modification and evolutionary trade-offs remain incompletely understood. *Salmonella* Pullorum is a pathogen that displays both O-antigen unstable phase variation and stable antigenic conversion between standard (O12_3_) and variant (O12_2_) antigenic types. In this study, we identified the glycosyltransferase encoded by *gtrC* ^III^ as responsible for the variant-specific glucosylation of the O-antigen. The expression of *gtrC* ^III^ is co-regulated by the transcriptional repressor OxyR and methyltransferase Dam, generating stochastic ON‒OFF switching of the O-antigen modification, which is a classic phase variation and promotes immune evasion. Remarkably, a single C-to-A point substitution at position –34 of the *gtrABC* ^III^ promoter disrupts OxyR binding and simultaneously enhances intrinsic promoter activity, resulting in constitutive expression of the variant antigenic type. This monophasic variant strain exhibits enhanced resistance to standard-type antibody-mediated killing, conferring a short-term immune evasion advantage. However, in long-term colonization, it is outcompeted by the phase-variable standard type strain, exemplifying “short-sighted evolution”, where an adaptive mutation trades off persistence for transient immune escape. Our findings reveal how a single nucleotide change can subvert a complex epigenetic switch to drive sub-serotype conversion, and provide insights into the evolutionary constraints shaping O-antigen diversity.

## Introduction

Bacterial pathogens continuously adapt to a range of challenges from the environment and host immune system, such as oxidative stress, antimicrobial peptides, bacteriophages, and antibody-mediated immunity. This adaptation drives both the evolutionary trajectory and regulatory architecture of bacterial virulence determinants.^1^ ^,^ ^2^ Virulence factors often need to be expressed in specific ecological niches, yet their excessive or untimely expression can impose substantial fitness costs, such as enhanced immune recognition.^3^ Such selective pressures also drive the microevolution of bacteria within the host, which enables bacteria to adapt to the local environment via short-term genetic mutations, phenotypic shifts, and reversible switches.^4^ While microevolution facilitates survival under acute selective pressures, certain adaptive mutations may compromise key features of long-term fitness such as transmissibility or virulence, a phenomenon termed “short-sighted evolution”.^5-7^ Understanding these evolutionary dynamics is critical for deciphering bacterial pathogenesis and informing bacterial surveillance and control strategies.

The O-antigen is located on the surface of Gram-negative bacteria and therefore is under intense selective pressure which makes it one of the most rapidly evolving bacterial structures.^8^ ^,^ ^9^ O-antigen biosynthesis is primarily governed by genes clustered in genomic islands, such as the *rfb* cluster in *Salmonella.* ^10^ ^,^ ^11^ Genetic variation in the O-antigen gene cluster is mainly mediated by horizontal gene transfer, genetic recombination, and lysogenic phages.^12-14^ Modifications to and structural variations of the O-antigen provide critical strategies for evading antibody recognition and other environmental stressors.^15^ ^,^ ^16^ Under long-term selective pressure, the O-antigen exhibits remarkable structural diversity due to variations in oligosaccharide repeat units, making it one of the most variable bacterial components and a primary determinant of serotype classification.^11^ ^,^ ^17^

In addition, different modifications, such as glycosylation and acetylation, further increase O-antigen structural heterogeneity.^18^ ^,^ ^19^ Glycosyltransferases (*gtr*), which are involved in the modification of O-antigen and serotype-specific conversion, have been identified in some bacteria, including *Shigella flexneri*, *Acinetobacter baumannii* and *Salmonella.* ^20-22^ Typically, the *gtr* operon comprises three genes, *gtrA*, *gtrB*, and *gtrC*. Among them, *gtrA* and *gtrB*, which encode the bactoprenol-linked glucosyltranslocase and bactoprenol glucosyltransferase, respectively, while *gtrC* is heterogeneous and acts as the serotype-specific glycosyltransferase.^23^ ^,^ ^24^ In *S*. Typhimurium, two *gtr* operons, have been well characterized. The *gtr* ^*p22*^ operon glycosylates the O-antigen, and confers additional recognition by O1 typing sera,^25^ while another *gtr* operon (STM0557-0559) introduces a 1-4-linked glucose, converting serovar O12 to O12_2._ ^19^ Expression analyses indicate that several *gtr* operons undergo phase variation, thus generating further O-antigen heterogeneity.^25^ ^,^ ^26^ Phase variation is an epigenetic regulation for controlling the switch in gene expression. This allows bacteria to generate phenotypically heterogeneous sub-populations within a genetically identical background, maintaining overall population fitness under various stress conditions.^27^ Beyond structural diversity, phase variation of surface antigen expression also importantly serves as a mechanism for immune evasion.^28^ ^,^ ^29^

**Figure 1.**
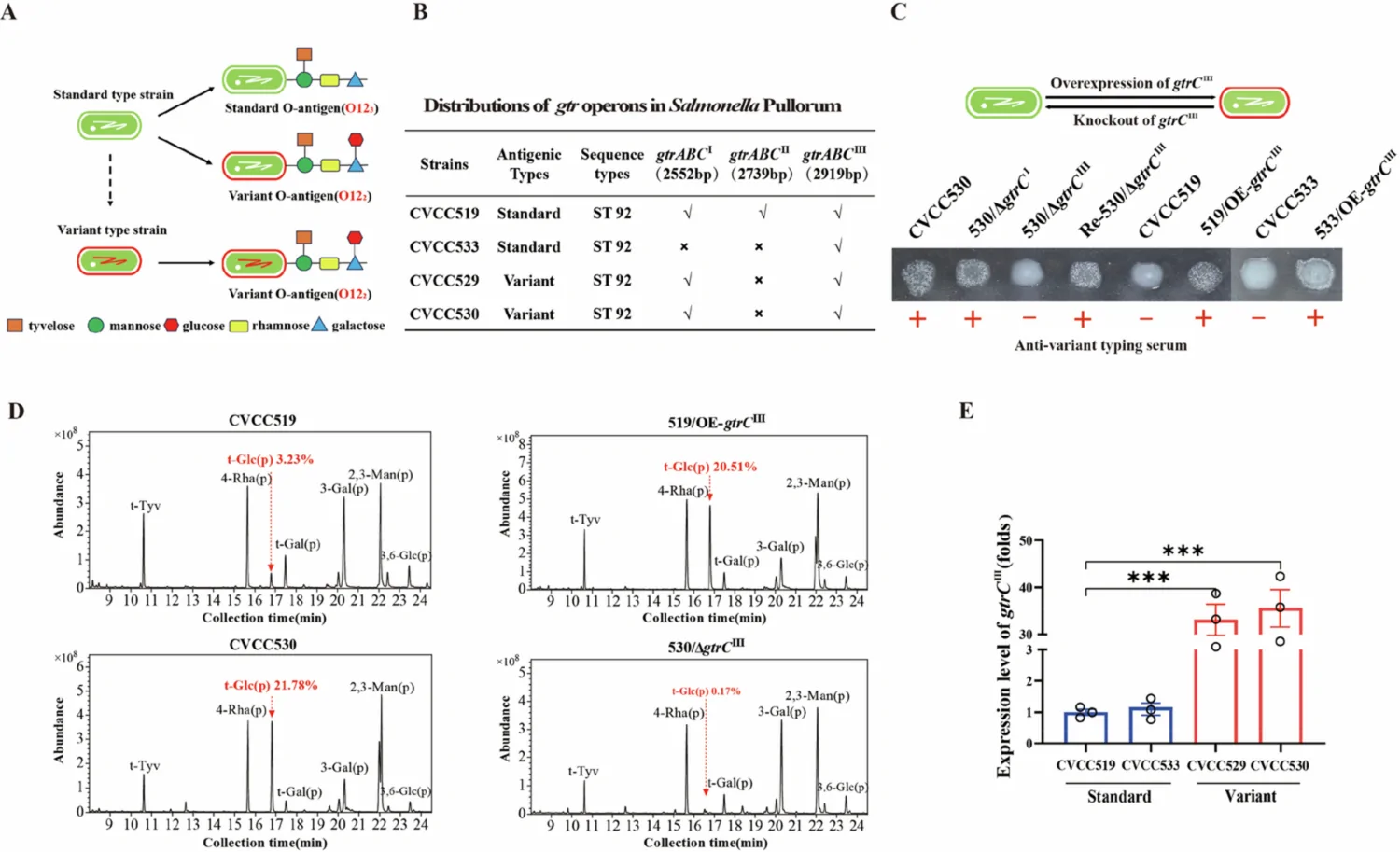
Identification of *gtrC* ^III^ as the glucosyltransferase responsible for the modification of O-antigen from standard type to variant type. (A) Schematic diagram of standard type and variant type *S*. Pullorum, and their O-antigen composition. Standard type strains can express two antigenic subtypes (O12_3_ and O12_2_), while variant type strains only express one antigenic subtype (O12_2_). (B) Distributions of *gtr* operons in the *S*. Pullorum standard type strains (CVCC519 and CVCC533) and variant type strains (CVCC529 and CVCC530). (C) The agglutination reactions of strains with anti-variant typing serum. The tested strains include variant type strain CVCC530 and its derivatives (530/Δ*gtrC* ^I^, 530/Δ*gtrC* ^III^ and Re-530/Δ*gtrC* ^III^), the standard type strain CVCC519 and its *gtrC* ^III^ overexpression strain 519/OE-g*trC* ^III^, and the standard type strain CVCC533 and its *gtrC* ^III^ overexpression strain 533/OE-g*trC* ^III^. The positive reactions were marked with “+”, and the negative reactions were marked with “–”. (D) GC‒MS linkage analysis of the O-antigen carbohydrates in CVCC530, 530/Δ*gtrC* ^III^, CVCC519 and 519/OE-g*trC* ^III^. Each peak represents a different carbohydrate, and the peak area represents the content. The peaks of T-Glc and its proportion in the total peak area (%) were marked with red frames. Among the four samples, Tyv, Rha and Man displayed similar high amounts, while the Gal content varied and showed a negative correlation with Glc. Glc is link to Gal through glycosylation. Therefore, the increase in Glc glycosylation may hinder the exposure of the Gal residues during monosaccharide determination. (E) Comparison of the expression levels of *gtrC* ^III^ in standard type strains (CVCC519 and CVCC533) and variant type strains (CVCC529 and CVCC530). Statistical significance was assessed using One-way ANOVA followed by Tukey's post-hoc test. Significance levels were defined as ns (not significant) and ***(*p* < 0.001).

*Salmonella enterica* serovar Gallinarum biovar Pullorum (*S*. Pullorum) is an avian-specific pathogen responsible for pullorum disease characterized by severe intestinal inflammation and white, sticky diarrhea, which can also lead to systemic infection. It causes high mortality in young birds and persistent chronic infection in adults.^30^ *S*. Pullorum has two distinct O-antigenic types: standard type (O12_3_) and variant type (O12_2_).^31^ Notably, the standard type strains exhibit O-antigen phase variation and also produce the O12_2_ variant type during subculture, usually at a rate of around 5% compared to O12_3_. In contrast, the variant type strains appear to be monophasic, lacking the capacity to express the O12_3_ standard type O-antigen (Figure 1A).^32^ This coexistence of phase variation and antigenic conversion makes *S*. Pullorum is a unique model for investigating the regulatory mechanisms and evolutionary drivers of O-antigen diversification in *Salmonella*. Previous efforts to link genomic differences to the two antigenic types of *S*. Pullorum using DNA fingerprinting failed to identify consistent discriminatory variations,^33^ suggesting that the distinction arises from subtle genetic or regulatory changes rather than large-scale genomic rearrangements. Recent structural analyses revealed that variant type LPS features an additional terminal glucose (T-Glc) linked to the C4 position of galactose in O-antigen repeats.^32^ However, the genetic basis for this modification and the mechanisms driving antigenic switching have remained unknown.

Here, we elucidate the molecular mechanism underlying O-antigen phase variation in standard type *S*. Pullorum and demonstrate its role in immune evasion. We first identified a single nucleotide polymorphism in the promoter of the glycosyltransferase operon that drives antigenic type conversion and distinguishes standard type from monophasic variant type strains. This mutation allows variant type strains to resist recognition by standard type specific antibodies, but imposes a fitness cost in long-term immune evasion and colonization, serving as the first characterized example of short-sighted microevolution in O-antigen. Collectively, our findings provide a foundation for the development of improved diagnostic tools and vaccines against *Salmonella* infections.

## Results

### Identification of the glycosyltransferase responsible for antigenic type conversion

Previous comparative analysis between *S*. Pullorum standard type strains (O12_3_) and variant type strains (O12_2_) revealed an additional glucosylation modification on the O-antigen in variant strains, which differentiates the O12_2_ variant^32^ (Figure 1A). Genomic analysis of *S*. Pullorum isolates and reference strains identified 1–3 *gtrABC* operons with three different combinations (*gtrABC* ^I^ + *gtrABC* ^II^ + *gtrABC* ^III^, *gtrABC* ^I^ + *gtrABC* ^III^, and *gtrABC* ^III^ only). We chose two standard type reference strains and two variant type reference strains as representatives of strains containing different combinations of *gtr* operons (Figure 1B). In the standard type strain CVCC519, three distinct *gtrABC* operons were found. Their sizes were 2552 bp (defined as *gtrABC* ^I^), 2739 bp (defined as *gtrABC* ^II^), and 2919 bp (defined as *gtrABC* ^III^), respectively. Another standard type strain, CVCC533, contained only *gtrABC* ^III^. In the variant type strain (CVCC529 and CVCC530), there were two *gtrABC* operons, *gtrABC* ^I^ and *gtrABC* ^III^ (Figure 1B).

To identify the specific glycosyltransferase gene responsible for producing the variant O-antigen O12_2_, we began by examining the *gtrC* genes in the monophasic variant type strain CVCC530 because it produces only the O12_2_ O-antigen type, making it a suitable starting point for investigation. The *gtrC* genes in *gtrABC* ^I^ and *gtrABC* ^III^ of the monophasic variant type strain CVCC530 were deleted separately (designated as 530/Δ*gtrC* ^I^ and 530/Δ*gtrC* ^III^, respectively). As shown in Figure 1C, the 530/Δ*gtrC* ^III^ mutant lost reactivity with anti-variant typing serum, while complementation (Re-530/Δ*gtrC* ^III^) restored agglutination. In contrast, the *gtrC* ^Ⅰ^ deletion had no effect on seroreactivity. GC‒MS linkage analysis of O-antigens from CVCC530 and 530/Δ*gtrC* ^III^ further confirmed the antigenic type conversion. As shown in Figure 1D and Table S3, the wild-type CVCC530 O-antigen contained abundant terminal glucose (T-Glc), and this modification was absent in 530/Δ*gtrC* ^III^. These results demonstrate that *gtrC* ^III^ is the key glycosyltransferase gene for producing the variant antigenic type.

Interestingly, *gtrC* ^III^ was previously identified as present in both standard and variant type strains (Figure 1B), with identical coding sequences, yet the majority of O-antigen in the standard type strains is O12_3_, whereas in the variant type strains, it is glycosylated O12_2_. We therefore hypothesized that differential *gtrC* ^III^ gene expression might cause this phenotypic variation. To test this, we used qRT-PCR and found that the expression levels of *gtrC* ^III^ in variant antigenic strains (CVCC529 and CVCC530) were approximately 30-fold higher than those in standard antigenic type strains (CVCC519 and CVCC533) (Figure 1E). To directly test whether *gtrC* ^III^ expression level determines the antigenic type, we constructed two *gtrC* ^III^ overexpression strains by using a constitutive strong promoter in standard type strains CVCC519 (519/OE-*gtrC* ^III^) and CVCC533 (533/OE-*gtrC* ^III^). Serum agglutination test showed that both 519/OE-*gtrC* ^III^ and 533/OE-*gtrC* ^III^ could agglutinate with anti-variant typing serum, indicating that they greatly increased their expression of the variant O-antigen (Figure 1C). GC‒MS analysis further confirmed increased T-Glc incorporation in 519/OE-*gtrC* ^III^ compared to wild-type CVCC519 (Figure 1D and Table S3). These findings demonstrate that upregulation of *gtrC* ^III^ expression drives the glucosylation modification that converts the standard to variant antigenic type.

### The differential expression of *gtrC* ^**III**^ is determined by the difference in promoter activity

**Figure 2.**
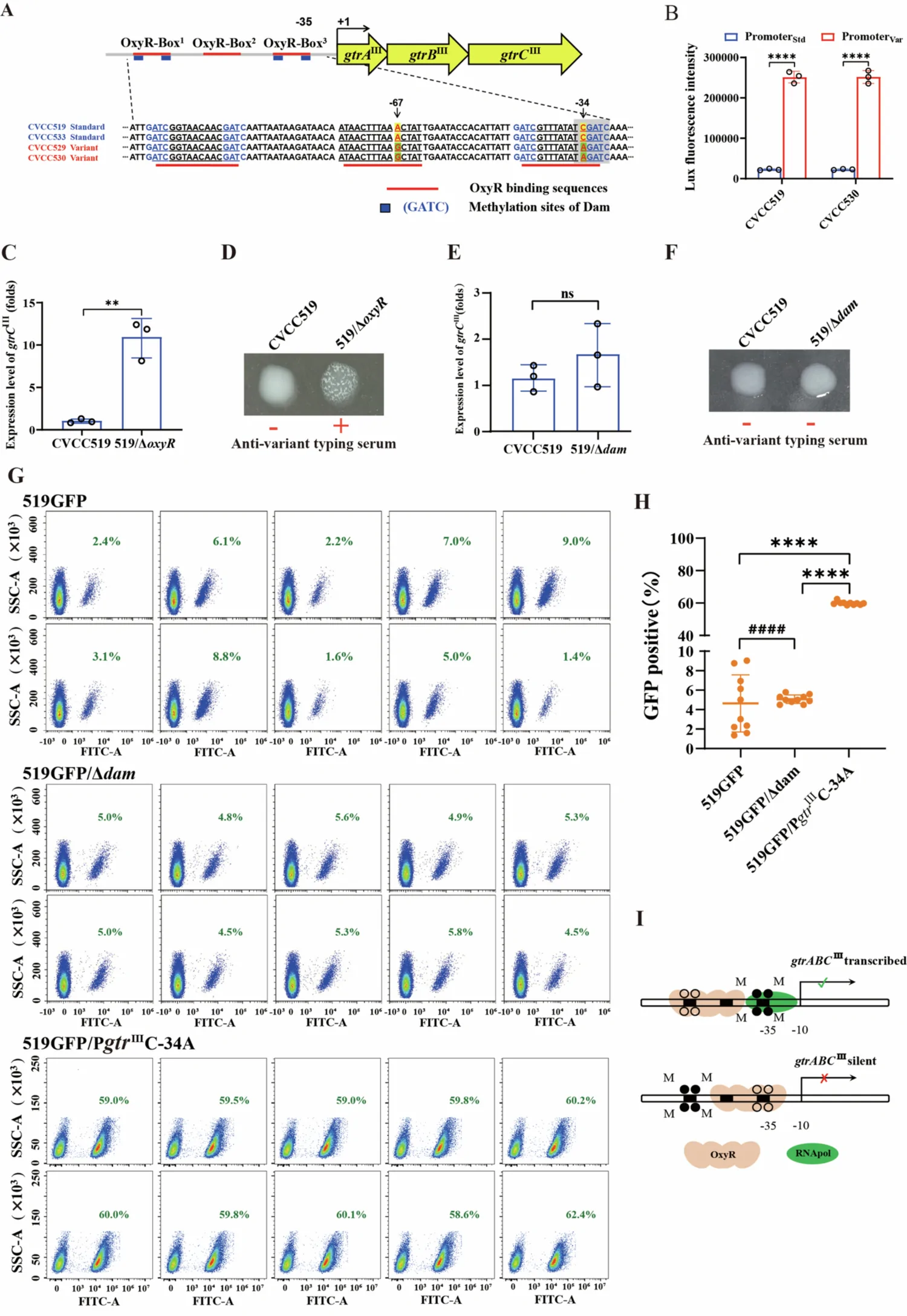
Co-regulation of OxyR and dam in the expression of *gtrC* ^III^. (A) The sequence analysis of the *gtrABC* ^III^ promoter in standard type strains (CVCC519 and CVCC533) and variant type strains (CVCC529 and CVCC530). The three OxyR binding sequences (OxyR-Box^1^, OxyR-Box^2^, and OxyR-Box^3^) are marked with a red underline, the blue letters (GATC) indicate the Dam methylation sites, and the red letters show the SNPs between standard and variant type strains. (B) Analysis of the Lux bioluminescence intensities controlled by different *gtrABC* ^III^ promoters under the CVCC519 or CVCC530 background. Statistical significance was assessed using Two-way ANOVA followed by Tukey's post-hoc test. Significance levels were defined as ns (not significant) and ****(*p* < 0.0001). (C) Comparison of the expression levels of *gtrC* ^III^ in CVCC519 and 519/Δ*oxyR* using Student's *t*-test. Significance level was defined as **(*p* < 0.01). (D) The agglutination reactions of CVCC519 and 519/Δ*oxyR* with anti-variant typing serum. (E) Comparison of the expression levels of *gtrC* ^III^ in CVCC519 and 519/Δ*dam* using Student's *t*-test. Significance level was defined as ns (not significant). (F) The agglutination reactions of CVCC519 and 519/Δ*dam* with anti-variant typing serum. (G) Analysis of the effect of *dam* deletion and −34 SNP on *gtrC* ^III^ expression in a subpopulation using single-copy *gtrABC* ^III^-GFP fusions. Ten bacterial colonies of each strain were picked from the plates and diluted in PBS, then the GFP fluorescence positive rates of 519GFP, 519GFP/Δ*dam*, and 519GFP/Pgtr^III^C-34A were detected using flow cytometry. The data are represented by a dot plot (side scatter versus fluorescence intensity [*gtrABC* ^III^ :*gfp* expression]). All the data were collected for 10,000 events per sample. (H) Comparison of GFP fluorescence positive rates and distributions among 519GFP, 519GFP/Δ*dam* and 519GFP/Pgtr^III^C-34A. The data are presented as individual values (*n* = 10). Levene's test was performed to evaluate differences in the data dispersion among the three groups. A significantly higher variance was observed in CVCC519 compared with the *dam* deletion mutant and Pgtr^III^C-34A SNP strain (^####^ *p* < 0.0001). The fluorescence positive rates were compared using one-way ANOVA followed by Tukey's post-hoc test. (****p < 0.0001). (I) Model for *gtrABC* ^III^ phase variable expression, referring to *gtrABC* (STM0557-0559) in *S*. Typhimurium.

Given that *gtrC* ^III^ coding sequences were identical between standard and variant strains, we next examined the promoter regions of the *gtrABC* ^III^ operon. Comparative analysis of *gtrABC* ^III^ promoter sequences from standard type and variant type strains revealed two conserved single nucleotide polymorphisms (SNPs): a C-to-A substitution at position –34 and an A-to-G substitution at position –67 relative to the transcription start site. There were four Dam methylation sites (GATC) and three transcriptional regulator OxyR binding sequences (OxyR-Box^1^, OxyR-Box^2^, and OxyR-Box^3^) within the *gtrABC* ^III^ promoter. Notably, the −67 SNP was located within OxyR-Box^2^, while the −34 SNP was located within OxyR-Box^3^ near a Dam methylation site (Figure 2A).

To investigate whether these promoter variations account for the differential *gtrC* ^III^ expression, we constructed promoter–Lux reporter systems using the *gtrABC* ^III^ promoters from CVCC519 (P_Std_) and CVCC530 (P_Var_) and introduced them into both CVCC519 and CVCC530 strains. As shown in Figure 2B, P_Var_ exhibited significantly higher expression activity than P_Std_ in both CVCC519 and CVCC530 backgrounds. This result suggested that promoter sequence variation determines the differential *gtrC* ^III^ expression between these antigenic type strains.

### Epigenetic co-regulation by OxyR and Dam controls phase-variable *gtrC* ^**III**^ expression in standard type strain

Given that the standard type strain exhibits O-antigen phase variation whereas the variant type does not, we next dissected the regulatory roles of OxyR and Dam in the standard type strain O-antigen phase variation. As a known transcriptional regulator, OxyR generally represses gene expression through promoter binding.^34^ *gtrC* ^III^ is expressed at a low level in standard type strain; therefore, we hypothesized that OxyR represses the expression of *gtrC* ^III^ in this context. To test this, we constructed an *oxyR* knockout in the standard type strain CVCC519 (519/Δ*oxyR*). The expression level of *gtrC* ^III^ was significantly increased in 519/Δ*oxyR* compared to CVCC519 (Figure 2C). At the same time, 519/Δ*oxyR* was agglutinative to anti-variant typing serum, indicating that it had undergone antigenic type conversion from standard to variant type with the loss of OxyR repression of the *gtrC* ^III^ glucosyltransferase, which we had implicated above as responsible for the addition of the T-Glc, which defines the switch from standard to variant O-antigen type (Figure 2D).

Dam methylation is known to generate stochastic phenotypic heterogeneity in other bacterial systems,^19^ we then investigated the role of Dam in the expression of *gtrC* ^III^ and thus O-antigen phase variation. We constructed a *dam* deletion strain in CVCC519 (519/Δ*dam*). As shown in Figure 2E and F, *dam* deletion neither altered *gtrC* ^III^ expression nor changed antigenic types at the population level. We then examined whether Dam contributes to phase variable expression of *gtrC* ^III^ at sub-population level in CVCC519. Single copy *gtrABC* ^III^:*gfp* translational fusions in the chromosome were constructed in the CVCC519 and 519/Δ*dam* backgrounds, and then, GFP expression were analyzed at single cell resolution using flow cytometry. As shown in Figure 2G and H, the GFP-positive sub-populations showed great heterogeneity in CVCC519, ranging from 1.36% to 9.04% of the total population, indicating stochastic variability in *gtrABC* ^III^ expression at a sub-population level. In contrast, the proportions of GFP-positive sub-populations in 519/Δ*dam*were all centered around 5% with minimal variations, showing loss of heterogeneity in *gtrABC* ^III^ expression in favor of population stability.

These data demonstrate that OxyR acts as the main regulator for controlling the expression of the *gtrABC* ^III^ operon, while Dam drives phenotypic heterogeneity by generating sub-populations with differential *gtrC* ^III^ expression, thereby enabling low frequency O-antigen phase variation in standard type strains. This epigenetic co-regulation pattern (three OxyR-Boxes, four methylation sites controlling by OxyR and Dam) is consistent with *gtrABC* (STM0557-0559) in *S*. Typhimurium.^9^ Referring to this study and our result, Figure 2I presents a graphical overview of our model for *gtrABC* ^III^ promoter regulation.

### A single C-34A substitution disrupts OxyR binding and enhances intrinsic promoter activity in variant type strain

**Figure 3.**
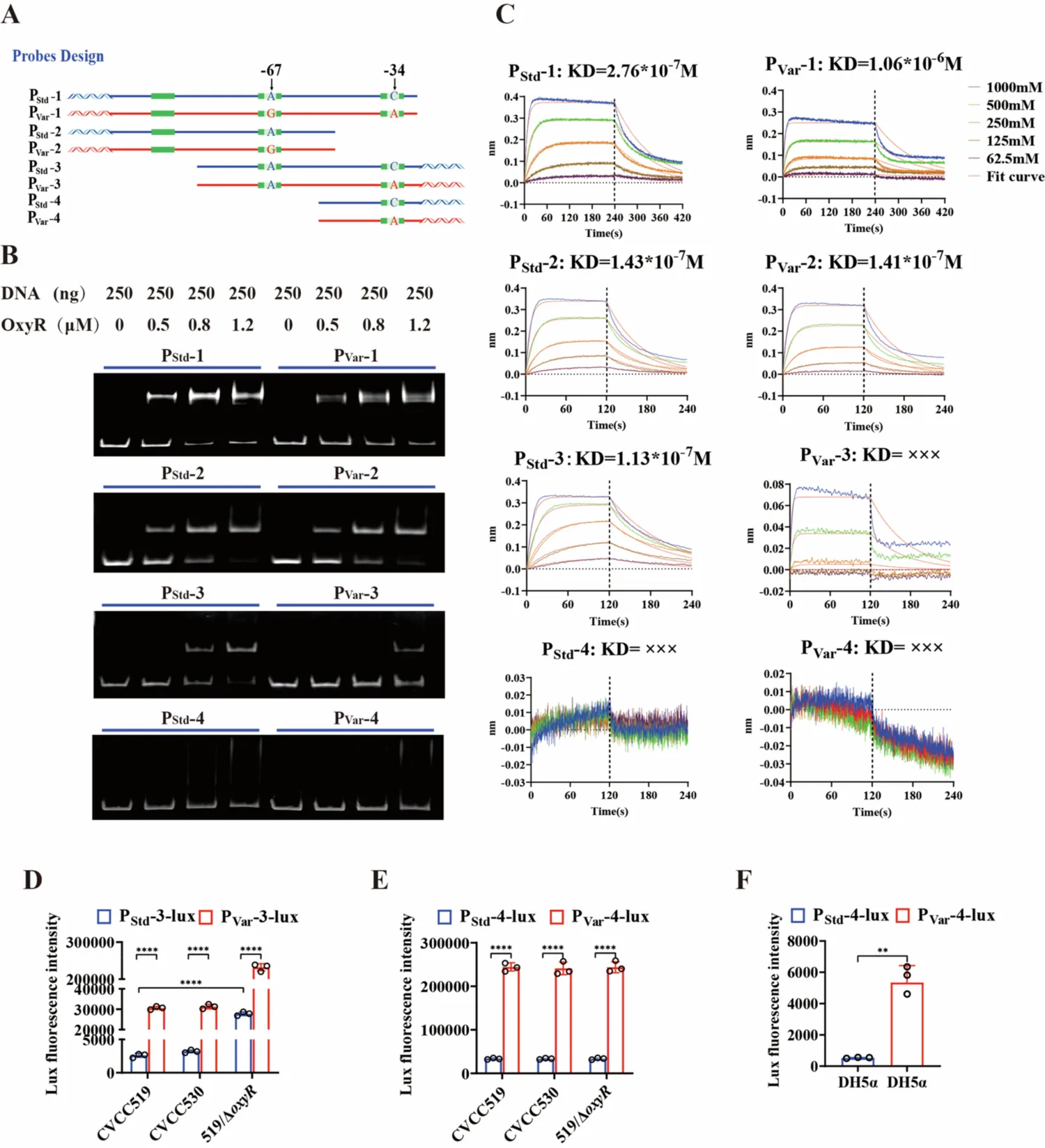
Identification of the key −34 SNP in the *gtrABC* ^III^ promoter and analysis of its effect on OxyR binding and *gtrABC* ^III^ expression. (A) Probe design for the *gtrABC* ^III^ promoter region. A total of 8 probes containing different combinations of OxyR-Box and SNPs were designed. OxyR-Boxes are shown in green. P_Std_-1 and P_Var_-1 were the full-length probes containing three OxyR-Boxes and both −34 site and −67 site SNPs, P_Std_-2 and P_Var_-2 contained the first two OxyR-Boxes and −67 site SNP, P_Std_-3 and P_Var_-3 contained the latter two OxyR-Boxes and −34 site SNP, and P_Std_-4 and P_Var_-4 contained one last OxyR-Box and −34 site SNP. (B) Electrophoretic mobility shift assay (EMSA) analysis of OxyR binding to the different promoter DNA probes. In this test, 0.25 μg promoter DNA probe and different amounts of purified recombinant OxyR^C199S^ protein (0, 0.5, 0.8, and 1.2 μM) were added. (C) Biolayer interferometry (BLI)-based assay of the binding of OxyR to different promoter DNA probes, as measured by affinity constants (KD values). (D) Comparison of the Lux bioluminescence intensities promoted by P_Std_-3 and P_Var_-3 in CVCC519, CVCC530, or 519/δ*oxyR* backgrounds. (E) Comparison of the Lux bioluminescence intensities promoted by P_Std_-4 and P_Var_-4 in CVCC519, CVCC530, or 519/δ*oxyR* backgrounds. (F) Analysis of the Lux bioluminescence intensities controlled by P_Std_-4 and P_Var_-4 in *E. coli* DH5α background. The statistical significance of the Lux bioluminescence intensities was determined by two-way ANOVA with Tukey's post-hoc test (****p < 0.0001, **p < 0.01).

In *S*. Typhimurium, OxyR acts as a tetramer to alternately bind two of three OxyR-Boxes. Binding of OxyR-Box^2^ and OxyR-Box^3^ leads to repression of gene expression.^9^ ^,^ ^25^ In our *S*. Pullorum strains, two SNPs between standard and variant type strains, were identified within the OxyR-Box of the *gtrABC* ^III^ promoter. To investigate the functional consequences of these polymorphisms, we analyzed the binding affinity of OxyR for various promoter constructs. Eight truncated or mutated promoter probes were used (Figure 3A). P_std_-1 and P_var_-1 contain all 3 Oxy-Boxes, whereas P_std_-2 and P_var_-2 contain Oxy-Boxes 1 and 2, P_std_-3 and P_var_-3 contain Oxy-Boxes 2 and 3, and P_std_-4 and P_var_-4 only contain Oxy-Box 3. P_std_ and P_var_ promoters express the divergent SNPs identified earlier at the −67 and −34 positions where applicable. Electrophoretic mobility shift assays (EMSAs) showed that while there were no obvious differences in OxyR binding affinity between P_Std_-1 and P_Var_-1 or P_Std_-2 and P_Var_-2, OxyR binding to P_Var_-3 was significantly reduced compared to P_Std_-3. However, OxyR binding to both P_Std_-4 and P_Var_-4 was nearly abolished (Figure 3B). BLI quantitative analysis further showed that the binding affinities of OxyR with P_Std_-2 (KD = 1.43 × 10^−7^ M) and P_Var_-2 (KD = 1.41 × 10^−7^ M) were comparable, while OxyR showed significantly weaker binding to P_Var_-3 (undetectable KD) compared to P_Std_-3 (KD = 1.13 × 10^−7^ M). Meanwhile, OxyR binding to P_Std_-4 and P_Var_-4, which contain only a single OxyR-box, was undetectable by BLI. According to previous reports, OxyR binds DNA as a dimer of dimers, thereby binding two OxyR-Boxes once.^35^ Our results also confirmed that OxyR binds two of three OxyR-Boxes in the promoter of *gtrABC* ^III^ and that two OxyR-Boxes need to be available for OxyR to successfully bind. Among the two SNPs found in the promoter, the SNP at position −67 in OxyR-Box^2^ does not affect OxyR binding, based on the comparable binding of OxyR to both Pstd-2 and Pvar-2. However, the “C” to “A” substitution at the −34 position in OxyR-Box^3^ significantly reduces OxyR binding affinity to the promoter region, likely by reducing to OxyR-Box^3^ and thus also to OxyR-Box^2^. This is important, as our results above demonstrated that OxyR binding played a central role in controlling the expression of *gtrABC* ^III^.

Our earlier results also found that native P_Var_-1 exhibited significantly higher expression activity than P_Std_-1 (Figure 2B). To further assess the functional impact of these sequence variations, we constructed promoter–Lux reporter systems using truncated promoters and introduced them into the CVCC519 and CVCC530 strains, with 519/Δ*oxyR* (which lacks OxyR-mediated repression) as a control. As predicted, truncated P_Var_-3 promoters drove higher levels of Lux expression in comparison to P_Std_-3 in both CVCC519 and CVCC530 (Figure 3D). Surprisingly, the expression of Lux driven by P_Var_-3 remained higher in 519/Δ*oxyR*, which lost OxyR repression, compared to P_Std_-3 (Figure 3D), suggesting that loss of OxyR binding was not the only factor driving enhanced promoter activity via the −34 variant SNP in P_var_. We further evaluated the activities of minimal promoters (P_Std_-4 and P_Var_-4), which contained only one OxyR-Box and thus completely lost their OxyR binding abilities. P_Var_-4 (which specifically contained the −34 “A” substitution in the remaining OxyR-Box^3^) maintained higher activity not only in tested *S*. Pullorum strains but also in *E. coli* (Figure 3E and F), suggesting intrinsic promoter-strengthening effects. The −34 site substitution resides within the −35-promoter element, a critical region for transcription initiation. Our findings suggested that this C-34A substitution not only reduces OxyR-mediated repression by diminishing transcription factor binding but also enhances intrinsic promoter activity.

**Figure 4.**
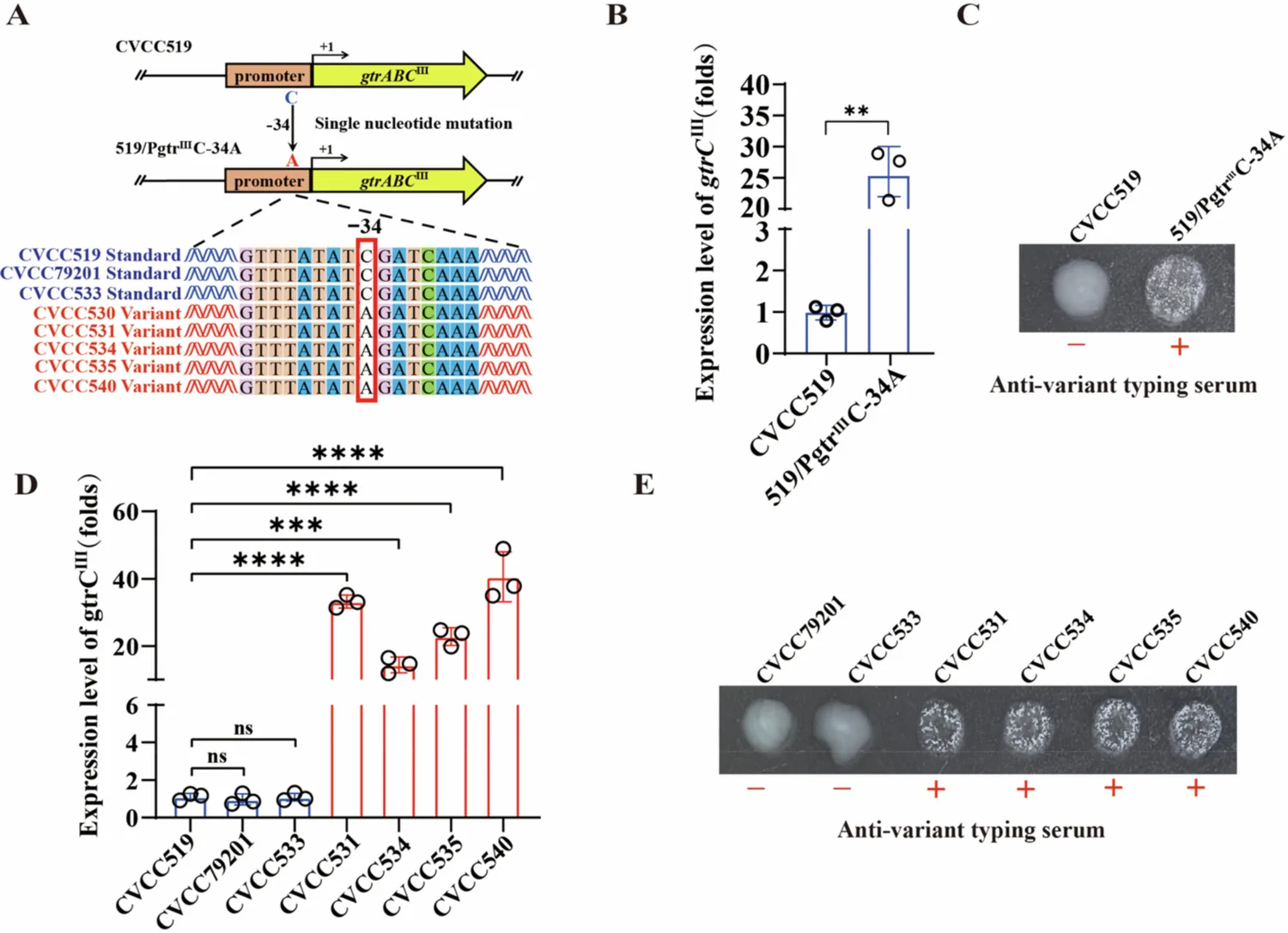
Confirmation of the role of the C-34A substitution in the *gtrABC* ^III^ promoter in antigenic type determinant. (A) Analysis of the −34 sites of *gtrABC* ^III^ promoters in additional standard and variant type reference strains. All the reference strains were obtained from China Institute of Veterinary Drug Control. CVCC79201 and CVCC533 were isolated from China, CVCC531 was isolated from the UK, CVCC534 and CVCC535 were isolated from United States, and CVCC540 was isolated from France. (B) Comparison of the expression levels of *gtrC* ^III^ in CVCC519 and 519/Pgtr^III^C-34A using Student's *t*-test. Significance level was defined as **(*p* < 0.01). Strain 519/Pgtr^III^C-34A was a C-34A point mutant derived from CVCC519. (C) The agglutination reactions of CVCC519 and 519/Pgtr^III^C-34A with anti-variant typing serum. (D) Analysis of the expression levels of *gtrC* ^III^ in the standard and variant type reference strains listed above. The *gtrC* ^III^ expression by each tested strain was compared with that of CVCC519 using Student's *t*-test. Significance levels were defined as ns (*p* > 0.05), ***(*p* < 0.001) and ****(*p* < 0.0001). (E) The agglutination reactions of the above reference strains with anti-variant typing serum.

To further confirm the functional role of the −34 site “C” to “A” point substitution in *S*. Pullorum O-antigenic switching, we introduced this specific point mutation into the standard type strain CVCC519 (519/Pgtr^III^C-34A) (Figure 4A). The 519/Pgtr^III^C-34A mutant exhibited a 25-fold upregulation of *gtrC* ^III^ expression compared to the parental CVCC519 strain (Figure 4B), and demonstrated positive agglutination with anti-variant typing serum (Figure 4C). The *gtrABC* ^III^:*gfp* translational fusion in 519/Pgtr^III^C-34A was also constructed (519GFP/Pgtr^III^C-34A) and analyzed using flow cytometry. The GFP-positive sub-populations of 519GFP/Pgtr^III^C-34A was centered around 60%, showing a significant increase compared to 519GFP (Figure 2G and H). This result indicated that the antigenic type of 519/Pgtr^III^C-34A has been switched from standard to variant type. We further analyzed a series of *S.* Pullorum strains with or without −34 site “C” to “A” point substitution (Figure 4A). All the strains harboring the “A” allele at the −34 position displayed both high *gtrC* ^III^ expression and anti-variant typing serum agglutination, whereas strains retaining the “C” allele showed low *gtrC* ^III^ expression and no agglutination with anti-variant typing serum (Figure 4D and E). These findings further demonstrate that the −34 site “C” to “A” substitution in the *gtrABC* ^III^ promoter alone is sufficient to convert standard to variant antigenic type. We screened 249 sequenced genomes in a systematic genomic study of *S*. Pullorum in China.^36^ This specific mutation was detected in only two isolates (SRAs: SRR19892354 and SRR19892370). This result was consistent with the fact that few variant type *S*. Pullorum isolates were identified in veterinary clinical practice recently.

### O-antigen phase variation occurs in the standard but not variant type strain *in vivo* and determines susceptibility to antibody-mediated clearance

**Figure 5.**
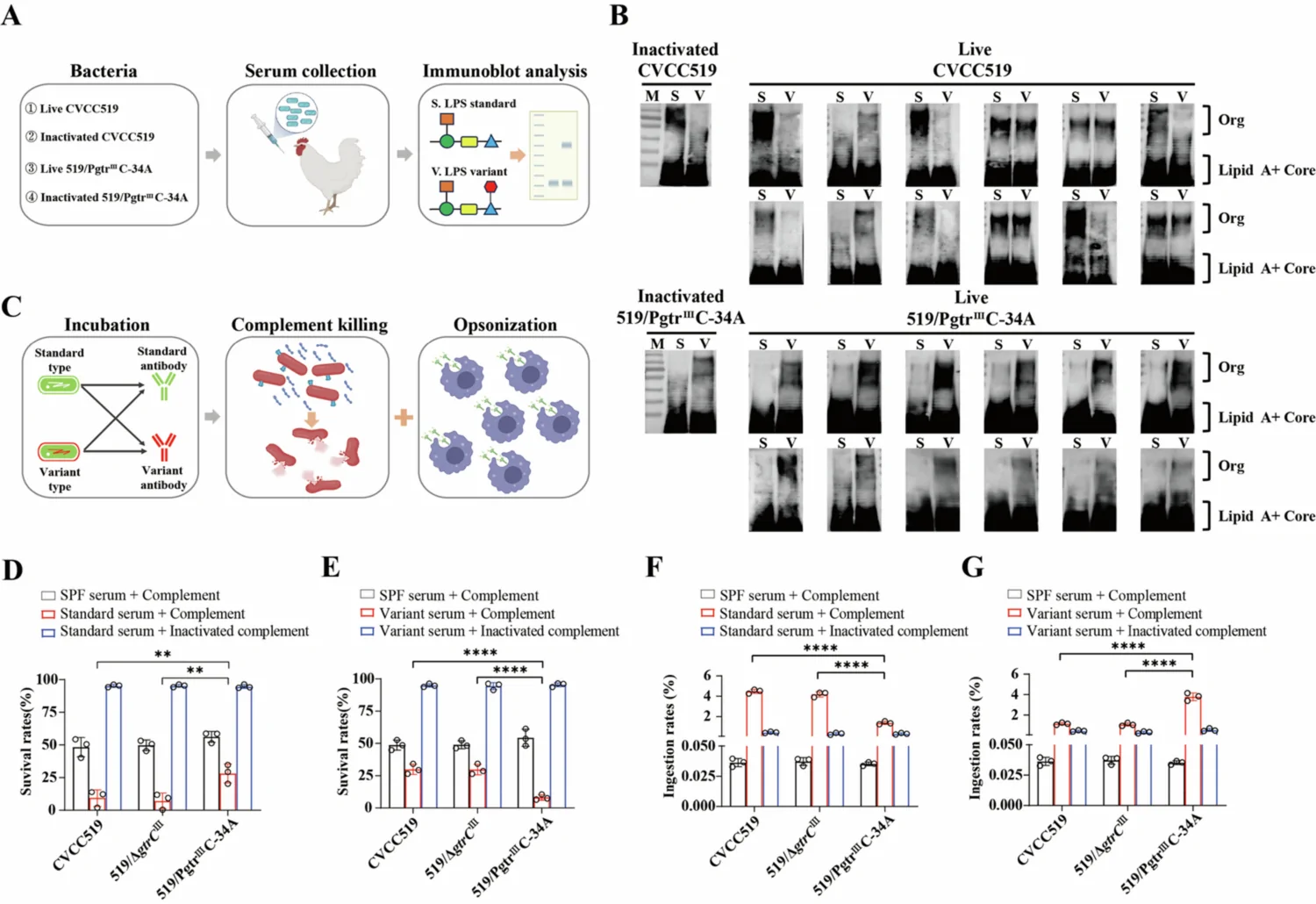
Analysis of serum recognition, antibody-dependent complement killing, and opsonophagocytosis. (A) Schematic diagram of serum recognition analysis. Chickens were inoculated with live CVCC519, inactivated CVCC519, live 519/Pgtr^III^C-34A, or inactivated 519/Pgtr^III^C-34A, respectively. Serum was collected at 28 d after inoculation. (B) Serum recognition of LPS from standard type strain (label S) and variant type strain (label V) was tested by Western blot. In live CVCC519 or 519/Pgtr^III^C-34A infected groups, sera from 12 individual chickens were used. The IgG antibody titers were tested using a *Salmonella* Group D Antibody test kit. Then, each serum was diluted to an identical titer (10^1^) and used for Western blot. (C) Schematic diagram of antibody-dependent complement killing and opsonophagocytosis analysis. (D and E) Antibody-dependent complement killing analysis. In each assay, the mixture contains 100 μL tested strain, 400 μL standard or variant antiserum (antibody component), and 500 μL fresh SPF chicken serum (complement component). (F and G) Opsonophagocytosis analysis. To opsonize the bacteria, the bacterial suspension was mixed with fresh or heat-inactivated specific serum and incubated at 37 °C for 30 min. Then, the opsonized bacteria were added to the HD11 cell (MOI = 100) for one hour to allow phagocytosis. The experiment was performed in three independent biological replicates using freshly plated HD11 cells. Control serum was collected from SPF chicken, while the standard or variant type sera were obtained from three independently immunized chickens. The data were analyzed by two-way ANOVA followed by Tukey's multiple comparisons test. *p*-values less than 0.05 were considered statistically significant, and the significance was marked as follows: ns (*p* > 0.05), *(*p* < 0.05), **(*p* < 0.01), ***(*p* < 0.001) and ****(*p* < 0.0001).

To analyze the occurrence of O-antigen phase variation during infection, we detected the production of specific antibodies in chickens infected with standard or variant type strains (CVCC519 and 519/Pgtr^III^C-34A). The antibody titers of different chickens were ranged from 10^3.0^ to 10^5.5^ but showed no significant difference between CVCC519 and 519/Pgtr^III^C-34A infected groups (Figure S2). Chickens immunized with inactivated CVCC519 or inactivated 519/Pgtr^III^C-34A strains were used as controls (Figure 5A). LPS recognition by serum was evaluated by Western blot. As shown in Figure 5B, chickens immunized with the inactivated CVCC519 strain produced serum that bound the standard O-antigen, whereas serum from chickens immunized with inactivated 519/Pgtr^III^C-34A reacted with the variant O-antigen, as expected. In the live 519/Pgtr^III^C-34A strain infection group, the results matched those of the inactivated 519/Pgtr^III^C-34A immunization: chickens mounted an anti-variant O-antigen response. In contrast, in the live CVCC519 strain infection group, serum reactions with O-antigen were heterogeneous. Among the serum samples collected from 12 individual chickens, six serum samples showed strong reactivity with standard O-antigen only, four serum samples had strong reactivity with both standard and variant O-antigens, and two serum samples only exhibited strong reactivity with variant O-antigen. In summary, live 519/Pgtr^III^C-34A infection primarily induced variant-specific antibody. In contrast, both standard- and variant-specific antibodies were induced in CVCC519-infected chickens. The presence of variant-specific antibodies in standard-type-infected chickens suggests that phase variation occurs *in vivo*, generating antigenic subpopulations that elicit distinct antibody responses.

Having established that different strains induced different host antibody responses to O-antigen, we then examined the functional properties of the antibody responses induced, beginning with antibody-dependent complement mediated cytotoxicity (Figure 5C). The incubation of standard type strains with standard type serum and complement resulted in significant lower bacterial survival rates (CVCC519, 9.23%, 519/Δ*gtrC* ^III^, 7.02%) in comparison to variant type strain (519/Pgtr^III^C-34A, 28.12%) (Figure 5D). Similarly, incubation of variant type strain with variant type serum and complement resulted in significant lower bacterial survival (519/Pgtr^III^C-34A, 7.94%) compared with standard type strains (CVCC519, 30.00%, 519/Δ*gtrC* ^III^, 29.78%) (Figure 5E). By contrast, no significant cytotoxicity was observed in the presence of heat-inactivated complement, while lower cytotoxicity (approximately 50%) was observed in the absence of a specific antibody (SPF serum), with no significant difference (*p*> 0.05) in survival rates between standard type and variant type strains (Figure 5D and E). These results demonstrate that O-antigen type-specific antibodies effectively trigger complement-mediated killing of their cognate strains, indicating robust activation of the classical complement pathway.

We then conducted phagocytosis assays to evaluate the opsonophagocytic activity of specific antibodies against standard and variant type strains (Figure 5C). Standard type antibody exhibited higher opsonic activity against standard type strains (CVCC519, 4.48%; 519/Δ*gtrC* ^III^, 4.19%) compared to the variant type strain (519/Pgtr^III^C-34A, 1.37%) (Figure 5F), while variant type antibody exhibited higher opsonic activity against 519/Pgtr^III^C-34A (3.78%) compared to CVCC519 (1.14%) and 519/Δ*gtrC* ^III^ (1.07%) (Figure 5G). Complement inactivation lowered the opsonic activity (to approximately 0.4%). Treatment with SPF serum instead led to an extremely low phagocytosis rate (approximately 0.04%) against all strains. These results demonstrate that standard type versus variant type-specific antibodies vary significantly in their capacity to promote opsonophagocytosis to *S*. Pullorum due to differences in epitope specificity.

### Loss of phase variation imposes a long-term colonization fitness cost

**Figure 6.**
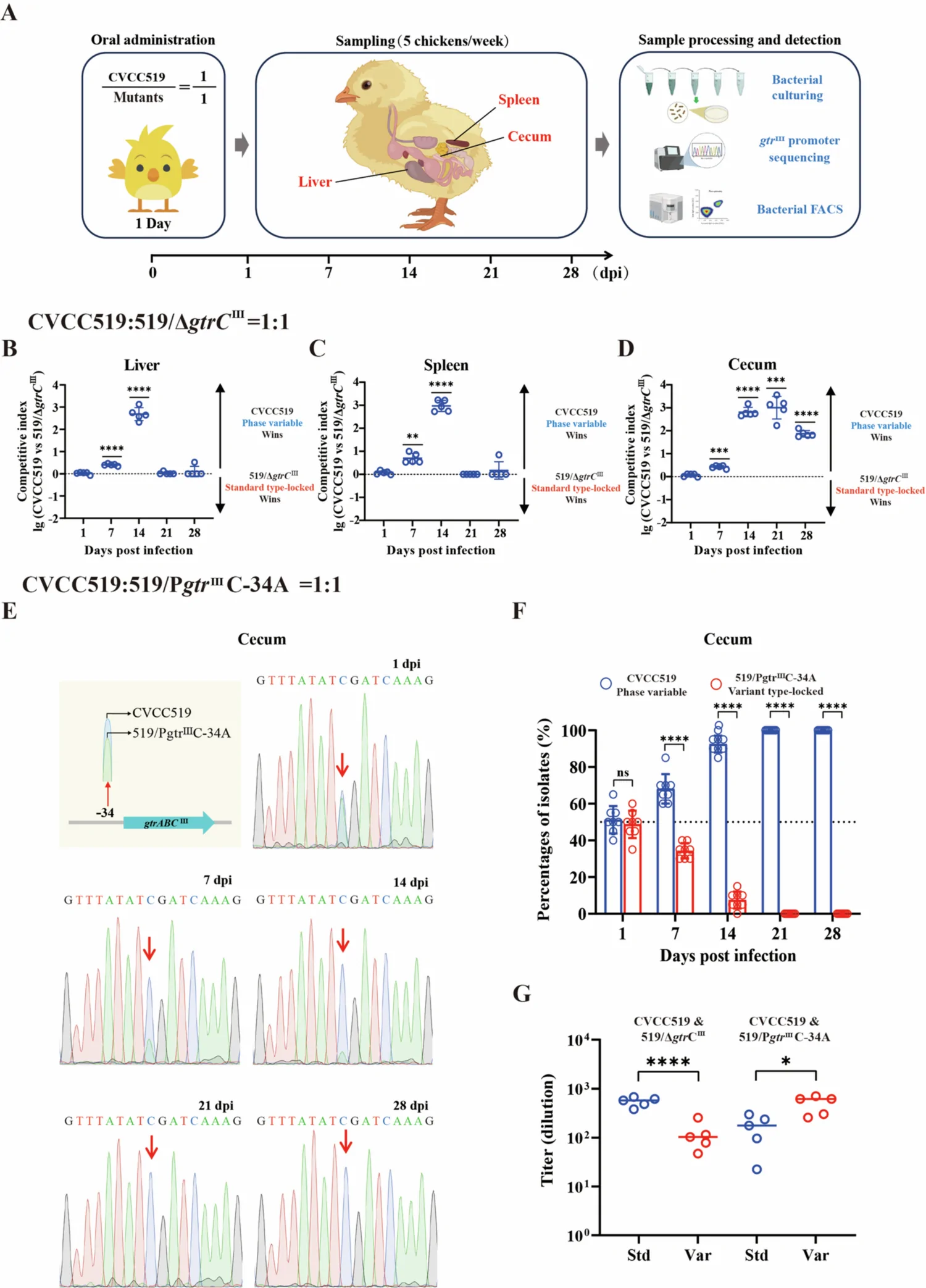
Competitive colonization assays of O-antigen phase variable strain and monophasic strain. (A) Schematic diagram of competitive colonization ability analysis. Each chicken was orally infected with a 1:1 mixture of CVCC519 and mutant (519/Δ*gtrC* ^III^ or 519/Pgtr^III^C-34A) and the bacterial burden was quantified at 1, 7, 14, 21, and 28 dpi. To further distinguish between CVCC519 and 519/Pgtr^III^C-34A, PCR targeting *gtrABC* ^III^ promoters and sequencing were used to discern the point mutation. (B–D) Competitive colonization abilities of CVCC519 and 519/Δ*gtrC* ^III^ in the liver, spleen, and cecum (*n* = 5). The competitive index (CI) was calculated by dividing the ratio of the two strains' loads in the tissues by their corresponding ratio in the inoculum. Statistical analysis was performed using One-way ANOVA followed by Tukey's post-hoc test for comparing the CI of each dpi with 0 dpi. **(*p* < 0.01), ***(*p* < 0.001), ****(*p* < 0.0001). (E and F) Comparison of the competitive colonization abilities of CVCC519 and 519/Pgtr^III^C-34A. The cecum contents were collected, and the promoter fragment of *gtrABC* ^III^ were amplified by PCR and sequencing. The sequencing peak maps revealed the relative abundance of each strain (E). To calculate the percentages of each strain, 100 single colonies on the plates were picked up and the promoter fragment of *gtrABC* ^III^ was further sequenced. The proportion of strains post-infection was analyzed by Student's *t*-test. ns (not significant), ****(*p* < 0.0001). (G) Titers of serum IgG specific for standard type strain (519/Δ*gtrC* ^III^) and variant type strain (519/Pgtr^III^C-34A), presented as the dilution of serum required to give an IgG-staining MFI = 1000 by bacterial flow cytometry. The sera were collected from chickens infected with a mixture of CVCC519 and 519/Δ*gtrC* ^III^ (*n* = 5), or mixture of CVCC519 and 519/Pgtr^III^C-34A (*n* = 5) at 14 dpi. Statistical significance was determined using Student's *t*-test. Significance level was defined as *(*p* < 0.05), ****(*p* < 0.0001).

An *in vitro* growth curve confirmed that there was no significant difference in the growth of CVCC519 versus 519/Δ*gtrC* ^III^ and 519/Pgtr^III^C-34A mutants in liquid culture (Figure S1). To evaluate the evolutionary consequences of losing phase variation, we performed competitive colonization experiments *in vivo*. Chickens were injected with 1:1 mixed CVCC519 strain (phase variable) and 519/Δ*gtrC* ^III^ (standard type-locked) or 519/Pgtr^III^C-34A (variant type-locked) mutant (Figure 6A). In the CVCC519 and 519/Δ*gtrC* ^III^ mixed infection group, CVCC519 became the dominant strain at 7 dpi and 14 dpi in the liver and spleen, demonstrating a significant colonization advantage compared to 519/Δ*gtrC* ^III^. However, by 21 dpi and 28 dpi, both CVCC519 and 519/Δ*gtrC* ^III^ were nearly eradicated in both the liver and spleen, negating any long-term competitive difference (Figure 6B and C). In the cecum, CVCC519 showed long-term persistence and colonization advantage compared to 519/Δ*gtrC* ^III^ (Figure 6D). In the CVCC519 and 519/Pgtr^III^C-34A mixed infection group, the colonization of bacteria in the cecum where the CVCC519 strain exhibited long-term persistence was detected. PCR and sequencing were used to distinguish CVCC519 (“C” at −34 site of *gtrC* ^III^ promoter) from 519/Pgtr^III^C-34A (“A” at −34 site). At 1, 7, and 14 dpi, there were twin peaks (“A” and “C”) at the −34 site, but then “A” was gradually decreased over time. At 21 and 28 dpi, “A” was no longer detected (Figure 6E). Corroborating this, 519/Pgtr^III^C-34A could not be isolated from the cecum at 21 and 28 dpi (Figure 6F). As above results, the bacteria began to exhibit the maximum colonization differences at 14 dpi. In order to analyze the correlation between specific antibodies and bacterial colonization, we detected specific antibody titers at this point. As shown in Figure 6G, in the CVCC519 and 519/Δ*gtrC* ^III^ mixed infection group, the titers of specific antibody against the standard strain were significantly higher than those against the variant strain and were negatively correlated with the colonization of the standard-locked 519/Δ*gtrC* ^III^ strain. In contrast, higher specific antibody titers against the variant strain were observed in the CVCC519 and 519/Pgtr^III^C-34A mixed infection group and were negatively correlated with the colonization of the variant-locked 519/Pgtr^III^C-34A strain. Therefore, the phase variable wild-type strain CVCC519 had a significant colonization advantage compared to both 519/Δ*gtrC* ^III^ and the 519/Pgtr^III^C-34A mutant.

These results demonstrate that despite variant-locked mutant can evade standard antibody-mediated killing, it was outcompeted by the phase-variable wild-type in long-term colonization *in vivo*. Thus, while the C-34A substitution confers a short-term advantage by evading standard type antibody-mediated killing, it imposes a long-term fitness cost by locking the bacterium in a single antigenic state, rendering it unable to adapt to shifting immune pressures—a hallmark of short-sighted evolution.

## Discussion

**Figure 7.**
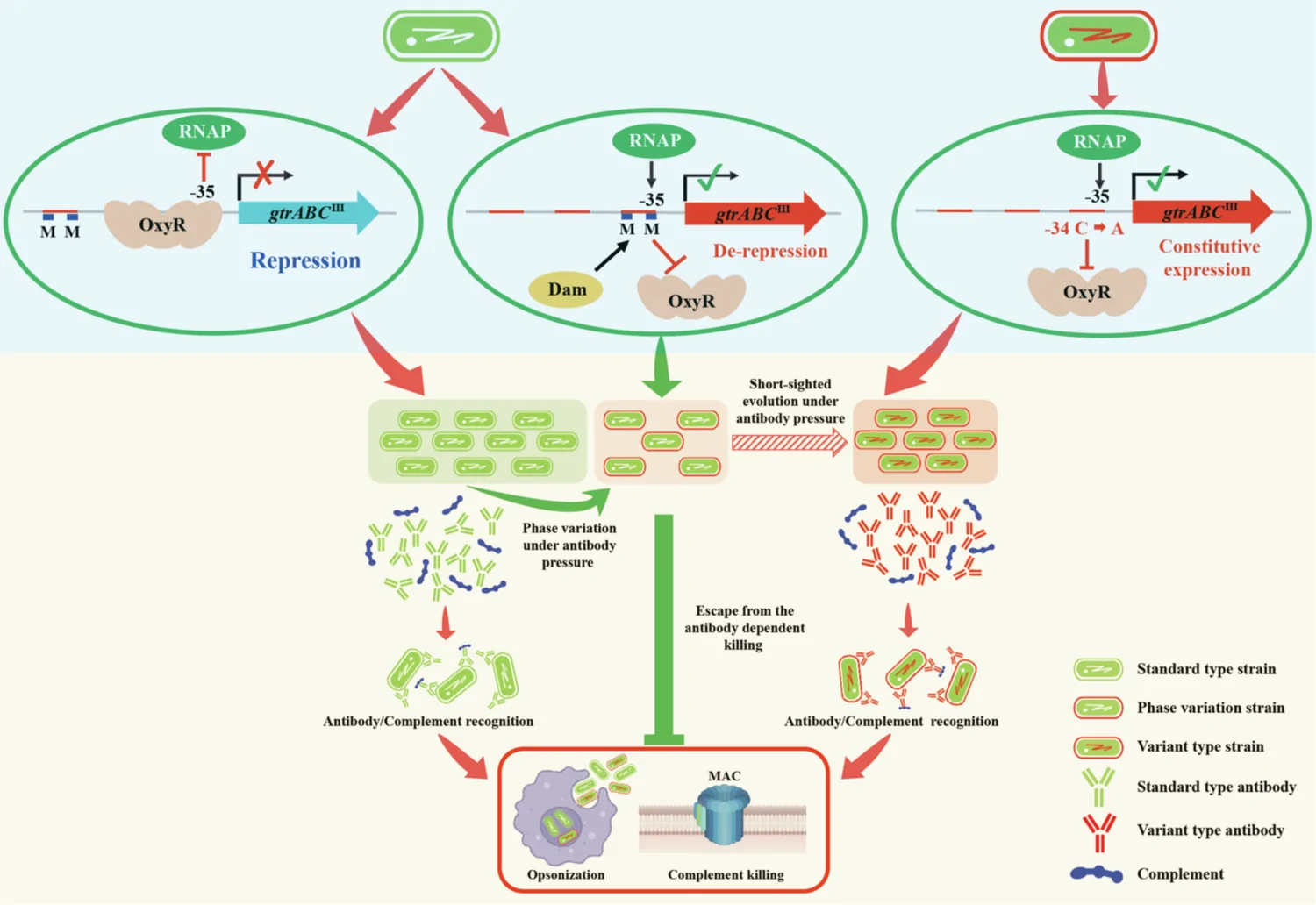
Proposed model of O-antigen phase variation and antigenic conversion in *S*. Pullorum and the effect of O-antigen dynamic modification on immune escape. The transcription factor OxyR and Dam methylase co-regulate the expression switch of *gtrABC* ^III^, resulting in the O-antigen phase variation of the standard type strain. The C-34A substitution leads to the de-repression and continuous high expression of *gtrABC* ^III^, forming a monophasic variant type strain. Under the pressure of the standard type antibody produced after infection, the phase variation of the standard type strain can evade the recognition of specific antibodies, thereby avoiding antibody-dependent complement killing and opsonophagocytosis. The variant type strain formed by the C-34A substitution is a “short-sighted evolution” under the pressure of standard type antibody, as it trades off its ability to evade the recognition of variant type antibody, and thus becomes easier for the immune system to clear over time.

*S*. Pullorum is a unique *Salmonella* serovar, in which antigenic conversion and phase variation between standard and variant antigenic types occur simultaneously, making it an ideal model for understanding O-antigen dynamics, as well as their effects on pathogenicity. In this study, we identified the *gtrC* ^III^ gene in *S.* Pullorum as the glycosyltransferase responsible for converting the standard O-antigen type (O12_3_) to the variant type (O12_2_) through the addition of a terminal glucose moiety. This finding resolves a long-standing question regarding the genetic basis of antigenic variation in this pathogen, which had remained elusive despite earlier efforts using DNA fingerprinting approaches.^33^ We demonstrate that the phase-variable expression of the *gtrABC* ^III^ operon is co-regulated by the transcriptional repressor OxyR and methyltransferase Dam, enabling reversible switching between the two antigenic states (termed O-antigen phase variation). Notably, a single C-to-A point substitution at the –34 site of the *gtrABC* ^III^ promoter abolishes this phase variation, resulting in a monophasic variant antigenic type. However, it seems a “short-sighted evolution” where an adaptive mutation trades off persistence for transient immune escape (Figure 7). To our knowledge, this represents the first demonstration that a single point substitution in a regulatory region can drive sub-serotype conversion in *Salmonella*.

The *gtrABC* ^III^ operon in *S*. Pullorum is homologous to the *gtr* operon (STM0557–STM0559) in *S*. Typhimurium, which is also responsible for adding glucose residues to repeating O-antigen subunits of LPS.^19^ Epigenetic co-regulation patterns, where Dam methylates the transcription factor binding site of promoters to cause the selective binding of transcription factors, have been found to control the expression of multiple virulence factors and antigens, such as ferritin-like protein (*ftnB*) and O-antigen chain length regulation factor (*opvAB*) in *Salmonella*,^37^ ^,^ ^38^ fumarate nitrate reductase in *Haemophilus influenzae*,^39^ and the membrane protein Agn43 in *E. coli.* ^40^ However, owing to the differences in the distribution of methylation sites and transcriptional binding sites, a wide variety of regulatory patterns are observed.^19^ In this study, the co-regulation pattern by OxyR and Dam identified here parallels that previously described for the *gtr* ^P22^ operon and the *gtr* operon (STM0557–STM0559) in *S*. Typhimurium.^19^ ^,^ ^25^ In this pattern (Figure 2I), three Oxy-Boxes exist in the promoter region of *gtrABC* ^III^, and OxyR binds to two of these three Oxy-Boxes. This OxyR binding of OxyR-Box^2^ and OxyR-Box^3^ significantly represses the expression of the *gtr* operon. Countering this, Dam-mediated methylation of GATC sequences in the OxyR-Box can inhibit OxyR binding to these sites, therefore altering *gtrABC* ^III^ operon transcription and generating stochastic heterogeneity in antigenic type expression at the single cell level. However, while *S*. Typhimurium and standard type *S*. Pullorum exhibit phase variation of the O12 antigen, the antigenic conversion from standard to variant type in *S*. Pullorum represents an additional unique feature, as it involves not only phase variation but also stable antigenic switching.

A central finding of this study is that a single C-to-A substitution at position –34 of the *gtrABC* ^III^ promoter is sufficient to abolish phase variation and lock the bacterium in the monophasic variant antigenic type. This mutation exerts a dual effect, in that it both reduces OxyR-mediated repression by disrupting OxyR-box^3^ binding and concurrently enhances the intrinsic activity of the promoter. Consequently, the *gtrABC* ^III^ operon is constitutively expressed, locking the bacterium in the monophasic variant O-antigen state. Our analysis of a panel of reference strains from several countries revealed a 100% correlation between the –34 allele and antigenic type, suggesting that this substitution is the primary determinant of the monophasic variant phenotype in *S*. Pullorum. More broadly, gene expression heterogeneity, which is jointly controlled by regulator and Dam has been identified in many bacterial pathogens, where it impacts the expression of virulence factors, antigens, and metabolic enzymes.^37^ ^,^ ^41^ Therefore, such mutations where a SNP in the core regulatory region of promoters may drive important phenotypic shifts in multiple phenotypes, including serotype, virulence, and metabolism in more bacteria. Our findings highlight how regulatory regions can serve as mutational hotspots that produce discrete phenotypic shifts, enabling rapid adaptation under selective pressure. These mutations may confer the formation of new serotypes and genotypes, and are worthy of attention in epidemiological monitoring.

Beyond its role in serotype determination, O-antigen glucosylation influences immune recognition. LPS is a potent immunogen, and antibodies directed against the O-antigen are a critical component of anti-pathogen adaptive immune responses.^42^ We showed that specific antibodies raised against the glucosylated or non-glucosylated O-antigen strongly recognize the matching LPS and significantly enhance antibody-mediated complement killing and opsonophagocytosis while exhibiting markedly reduced activity against mismatched strains. The classical complement pathway, which is initiated by antibody binding to surface antigens, importantly culminates in the formation of the membrane attack complex and direct bacterial lysis amongst other outcomes.^43^ Concurrently, opsonophagocytosis mediated by Fc receptor engagement on macrophages and neutrophils represents a second critical arm of antibody-dependent clearance.^44^ ^,^ ^45^ Therefore, the precise antibody recognition of the specific O-antigen glucosylation modification of *Salmonella* plays a crucial role in efficient immune clearance by the host.

O-antigen phase variation allows the bacterial population to dynamically alter its antigenic composition, providing a mechanism to evade antibody-mediated clearance. Knockout of Dam, the key regulatory factor of phase variation, has previously been reported to significantly reduce the inflammatory response to and virulence of *Salmonella*.^46-48^ Unlike a fixed antigenic state, phase variation generates a heterogeneous population in which a subset of cells expresses an alternative antigenic form.^25^ ^,^ ^28^ This strategy enables the bacterial population to escape from specific antibodies and persist under immune pressure. Our competitive colonization experiments support this interpretation. Wild type CVCC519 (O-antigen phase variable) exhibited a stronger long-term colonization ability, compared with the 519/Δ*gtrC* ^III^ mutant (O-antigen glucosylation incapable) and 519/Pgtr^III^C-34A mutant (O-antigen glucosylation continuous). We propose that during infection with a phase-variable strain, the host initially produces antibodies targeting the predominant standard O-antigen type. As infection progresses, the dominant antigenic type strains will also undergo changes under the pressure of specific antibodies. The diversity of serum recognition of different LPS after CVCC519 infection provided direct evidence for the changes in the dominant O-antigen subtype strains during this process (Figure 5B). This dynamic interplay between antigenic switching and antibody pressure likely underlies the long-term colonization advantage of phase variable strains.

Single-base mutations are one of the major drivers of bacterial evolution within the host. Under selective pressures from the host and environment, adaptive mutations can be retained. We inferred that the monophasic variant antigenic type strain is a result of microevolution to escape early antibody-mediated effects such as classical complement activation and opsonophagocytosis during chronic infections of the original standard antigenic type strain. However, the loss of phase variation by the variant antigenic type results in a trade-off that reduces competitive colonization fitness during chronic infection. We interpret this as a manifestation of “short-sighted evolution”, defined as a mutation that confers short-term immune evasion but compromises long-term fitness and transmissibility.^4^ ^,^ ^7^ The concept of short-sighted evolution was originally proposed to explain adaptations in pathogens that enhance survival within the current host at the expense of transmission to new hosts. In our system, when the bacterium faces a specific antibody response directed against the standard antigenic type during the early stages of infection, the –34 site promoter substitution provides a selective advantage under specific antibody pressure. However, the bacterium loses its capacity to subsequently switch away from the latter antibodies raised to target variant type O-antigen. Consequently, the mutant strain exhibited reduced colonization fitness over extended time courses. Indeed, only a small portion of the strains are variant antigenic type strains amongst clinical *S*. Pullorum isolates,^31^ ^,^ ^49^ which also supports that this is a short-sighted evolution. Analogous examples of short-sighted evolution have been documented in other chronic infections. For example, virulence-enhancing variants selected within the host are underrepresented in transmission chains in *Mycobacterium abscessus.* ^2^ In *Pseudomonas aeruginosa* during cystic fibrosis lung infections, some mutations confer antimicrobial resistance but often come with fitness costs in dissemination.^50^ Our study provides strong evidence for short-sighted evolution in the O-antigen of *S.* Pullorum.

In addition, our findings have practical implications for *Salmonella* diagnostics and vaccine development. Phase-variable O-antigen expression complicates the preparation of agglutination antigens for pullorum disease diagnosis, as the proportion of standard and variant components can vary between batches, leading to inconsistent diagnostic sensitivity. Understanding the regulatory basis of this variation offers a theoretical basis to in future design strains with stabilized antigenic properties, such as knockout or overexpression of the glycosyltransferase to lock the modification. In vaccine development, inactivated vaccines produced from *in vitro* cultures may not reflect the antigenic profiles that bacteria adopt *in vivo*, potentially limiting vaccine efficacy. A deeper understanding of the mechanisms governing O-antigen conversion and phase variation could inform the rational design of live attenuated vaccines that maintain immunogenic stability during infection.

Collectively, this study elucidates the molecular mechanism of O-antigen phase variation in *Salmonella*. We characterize a single point substitution as a driver of sub-serotype conversion, and provide an example of short-sighted microevolution in host adaptation. These insights contribute to a deeper understanding of *Salmonella* pathogenesis, which can inform strategies for improved disease control.

## Materials and methods

### Strains used in this study

The bacterial strains used in this study are listed in Table S1. All the mutants of *S.* Pullorum strains were derived from the CVCC519 or CVCC530 strains, which were obtained from the China Institute of Veterinary Drug Control. The strains were grown at 37 °C in Luria–Bertani (LB) broth. When required, 50 μg/ml streptomycin, 50 μg/ml kanamycin, 50 μg/ml chloromycetin, and 100 μg/ml ampicillin were added to the culture medium.

### Strains construction

All primers used for strain construction are listed in Table S2. Single gene deletion mutant strains, including Δ*gtrC* ^I^, Δ*gtrC* ^III^, Δ*oxyR* and Δ*dam*, were constructed using the λRed system as previously described.^51^ In brief, the chloramphenicol resistance cassette (*cat*) was amplified from the plasmid pKD3 by PCR, using primers that contained the homologous recombination arms of the target gene. The amplified *cat* was transformed into the parent strain containing plasmid pKD46. The target gene was replaced with the *cat* gene, and then the *cat* gene was removed using the plasmid pCP20, generating the mutant. For construction of the “C” to “A” point mutation in the CVCC519 strain, double recombination was carried out using the suicide plasmid pRE112 system.^52^ First, pRE112 carrying homologous arms were transferred into the strain, and the *gtrA* ^III^ gene and its promoter were deleted by homologous recombination. Then, the fragment containing the *gtrA* ^III^ gene and the mutant promoter (“C” to “A”) were inserted between two homologous arms of pRE112 and transferred into the mutant strain, and this fragment was inserted into the original site of the strain through the second homologous recombination. The point mutant was then confirmed by sequencing. For the construction of *gfp* transcriptional fusions, pRE112 carried homologous arms, and a promoter-less *gfp* gene was constructed. Through homologous recombination, a single copy of the *gfp* gene was inserted and fused with *gtrC* ^III^ downstream of this gene. For the construction of promoter–Lux reporter systems, pACYC^53^ plasmid derivatives carrying different truncated promoters and *lux* gene fusions were constructed and transferred into *S*. Pullorum or *E. coli* strains. For overexpression of *gtrC* ^III^ in the *S*. Pullorum strains, the ORF of *gtrC* ^III^ was cloned into the pCDF-J23119 plasmid (a gift from Rui Zhou's laboratory, Huazhong Agricultural University, China) and transferred into the tested strains. The expression of *gtrC* ^III^ was driven by the strong constitutive promoter J23119.

### Extraction of LPS and GC-mass spectroscopy

LPS was extracted from 2 L of bacterial culture using a modified hot-phenol water method.^54^ To prepare O-polysaccharides, LPS was treated with 1% acetic acid at 100 °C for 1 h, and the carbohydrate component was separated from lipid A. The glycosyl composition and glycosyl linkage analysis were performed at Huaster Biology (Wuhan, China) as previously described.^55^ The composition of each carbohydrate portion and the linkages of each carbohydrate were determined by GC–MS analysis based on the CCRC Spectral Database (https://glygen.ccrc.uga.edu/ccrc/specdb/ms/pmaa/pframe.html).

### Anti-serum agglutination test

Reference antisera were purchased from China Institute of Veterinary Drug Control (Bei Jing, China). Antigenic types were determined by serum agglutination tests. In brief, colonies were selected from modified Martin Agar plates and re-suspended in normal saline, the concentration of the bacterial suspension was adjusted to 1.0 × 10^10^ CFU/mL, and 25 μl of standard or variant antiserum and 25 μl of bacterial suspension were mixed on a glass plate and shaken gently. The agglutination reaction was observed within 2 min.

### Electrophoretic mobility shift assays (EMSA)

As previously reported, OxyR needs to exert its DNA-binding function in the oxidized state, and C199S amino acid substitution can simulate this oxidized state.^35^ A fragment containing *oxyR* ^C199S^ was cloned into pET28a (Novagen, Shanghai, China) and transferred into *E. coli* BL21 (DE3). The His-OxyR^C199S^ protein was expressed and purified using Ni-NTA agarose (Bio-Rad, USA) under native conditions according to the manufacturer's instructions. Eight probes containing different OxyR-boxes and point mutations were used (Figure 3A). His-OxyR^C199S^ binding reactions were carried out in a 20-μl volume containing the binding buffer (20 mM Tris–HCl, pH 8.0; 50 mM KCl; 5% glycerol; 0.5 mM DTT; 25 μg/ml BSA, 100 ng poly dIdC), 0.25 μg promoter DNA, and different amounts of purified recombinant OxyR^C199S^ protein (0, 0.5, 0.8, and 1.2 μM). The binding reaction was incubated at room temperature for 15 min. The electrophoresis was carried out with 5% non-denaturing gels, and then the gels were stained with ethidium bromide and visualized.

### Bio-layer interferometry (BLI)

BLI was used to further test the ability of OxyR^C199S^ protein to bind different mutant or truncated *gtrABC* ^III^ promoters. In brief, different biotin-labeled promoter probes were generated by PCR amplification using biotin-modified primers. Binding assays were performed in 96-well microplates using the Octet Red system (ForteBio, USA). Briefly, streptavidin (SA) sensors were coupled with biotinylated DNA probes (100 nM) in 200 μL PBS. Then, the coupled SA sensors were incubated with a two-fold serial dilution of OxyR^C199S^ protein (1000, 500, 250, 125, 62.5 mM) in HEPS buffer for 120 s at 30 °C, followed by a dissociation phase in buffer alone for 120 s. Affinity constants (KD values) were processed automatically using the Octet User Software version 5.0.

### Real-time RT-PCR

The primers used in real-time RT-PCR are listed in Table S2. Overnight cultured bacteria were diluted 1:100 in LB and then incubated at 37 °C to the mid-log phase (OD_600_ = 0.5). Total RNA was isolated and purified using the SV Total RNA Isolation System (Promega, USA) according to the manufacturer's instructions. The transcripts of the target genes were assessed by real-time RT-PCR using SYBR Green (Takara, China), with *gapdh* serving as the internal control. Differences in relative transcript abundance levels were calculated using the 2^–ΔΔCT^ method. Three biological replicates were performed for each test.

### Lux bioluminescence intensity detection

Bacteria containing the promoter–Lux reporter system were grown to the mid-log phase (OD_600_ = 0.5). The cells were harvested by centrifugation and washed three times with phosphate-buffered saline (PBS). The concentration of each bacterial suspension was then adjusted to an OD₆₀₀ of 0.5. The bacterial suspensions were transferred to a black microplate, and the bioluminescence intensity was measured using a microplate reader with an excitation wavelength of 584 nm and an emission wavelength of 607 nm. A strain that had not been transformed with the lux reporter gene was used as a blank control for background correction. Three independent biological replicates were performed for each experimental group, with each biological replicate comprising at least three technical replicates.

### Analysis of GFP expression by flow cytometry

Flow cytometry was used to monitor the expression of transcriptional *gtrABC* ^III^-GFP fusions in CVCC519. Flow cytometry was conducted on a CytoFLEX S flow cytometer (Beckman Coulter, Inc., Brea, California, USA). The fluorescence signals were collected and analyzed using the CytExpert acquisition and analysis software (version 2.4, Beckman Coulter). Ten bacterial colonies of 519GFP or 519GFP/Δ*dam* cultures were washed and re-suspended in PBS for fluorescence measurement. The fluorescence values for 10,000 events were compared with the data from the reporter-less control strain CVCC519, thus yielding the fraction of ON versus OFF cells.

### Western blot analysis

Polyvalent antisera were raised in SPF chickens immunized with inactivated CVCC519 or 519/Pgtr^III^C-34A strains or infected with live CVCC519 or 519/Pgtr^III^C-34A strains. For Western blot analyses, LPS from each strain was separated on a Tricine–SDS-PAGE gel and transferred onto a polyvinylidene difluoride (PVDF) membrane and blocked with 5% milk in PBS-Tween. Polyvalent antiserum was used as the primary antibody. The baseline IgG antibody titer of each serum was tested using a *Salmonella* Group D Antibody test kit (BioChek, The Netherlands) and subsequently diluted to identical titers (10^1^). The goat anti-chicken IgG-horseradish peroxidase (HRP) was used as the secondary antibody (bs-0310G, Bioss, China). Luminato Western HRP substrate (Millipore) was used for detection.

### Serum sensitivity assay

To generate standard and variant-specific antibody pools, antisera were raised in SPF chickens immunized with inactivated CVCC519 or 519/Pgtr^III^C-34A. Complement was obtained from the serum of freshly collected SPF chicken blood. For the serum-killing assays, 100 μL from each tested strain was collected at the mid-log phase (OD_600_ = 0.6), washed twice with PBS, and then added into the mixture containing 400 μL standard or variant antiserum (antibody component) and 500 μL fresh SPF chicken serum (complement component). The mixture was incubated for 1 h at 37 °C, following which dilutions were performed to determine the CFU. SPF serum was used as the antibody-free control, and heat-inactivated serum was used as the complement-free control. Bacterial survival was quantified relative to that of a PBS-treated control. Each sample was assayed in duplicate. The data represent at least three independent experiments.

### Opsonophagocytosis assay

A chicken macrophage cell line (HD11) was used for the opsonophagocytosis assay. Prior to infection, HD11 cells were washed twice with PBS and maintained in serum-free DMEM. Bacteria were grown to mid-log phase, harvested, and resuspended in PBS to a concentration of 1 × 10^8^ CFU/mL. To opsonize the bacteria, 200 µL of this bacterial suspension was mixed with 800 µL of fresh or heat-inactivated chicken serum and incubated at 37 °C for 30 min. Control serum was collected from SPF chickens. Standard type sera were obtained from three independent chickens immunized with inactivated 519/Δ*gtrC* ^III^. Variant type sera were obtained from three independent chickens immunized with inactivated 519/Pgtr^III^C-34A. Following opsonization, the bacteria were added to the HD11 cell monolayers at a multiplicity of infection (MOI) of 100 and co-incubated for 1 h at 37 °C to allow for phagocytosis. To kill any remaining extracellular bacteria, the cells were treated with DMEM containing 100 µg/mL penicillin for 1 h at 37 °C. The monolayers were then washed twice with DMEM to remove the antibiotic and lysed with 500 µL of ice-cold sterile water. The number of phagocytosed (internalized) bacteria was quantified by performing serial dilutions of the cell lysates and plating them on LB agar. Each condition was performed in duplicate. The data are representative of at least three independent experiments.

### Competitive colonization analysis

To evaluate the competitive colonization of bacteria that lost their capacity for phase variation, chickens were orally injected with a 1:1 mixture of CVCC519 and either 519/Δ*gtrC* ^III^ or 519/Pgtr^III^C-34A mutants. The total dose of bacteria was 2 × 10^8^ CFU per chicken (1 × 10^8^ CFU per strain). In the CVCC519 and 519/Δ*gtrC* ^III^ mixed infection group (*n* = 5), bacterial colonization was assessed in the liver, spleen, and cecum at 1, 7, 14, 21, and 28 d post-infection (dpi). The competitive index (CI) was calculated by dividing the ratio of the two strains' loads in the tissues by their corresponding ratio in the inoculum. Statistical analysis was performed using Student's *t*-test by comparing the CI of each dpi with 0 dpi. In the CVCC519 and 519/Pgtr^III^C-34A mixed infection group, only bacterial colonization in the cecum was monitored because CVCC519 mainly exhibited a longer period of colonization in the cecum. The mutation at the −34 site was assessed by PCR amplification followed by Sanger sequencing. The presence of single nucleotide polymorphisms (twin peaks “A” and “C”) was evaluated at different time points. To further confirm the result, 100 bacterial colonies were recovered, and the promoter site was sequenced to distinguish the two strains from each cecal sample. Colonization advantages were determined by comparing the recovery rates of CVCC519 versus the mutant strains at each time point.

### Analysis of specific antibody titers by bacterial flow cytometry

To analyze the correlation between specific antibodies and colonization, the specific antibody titers in the infected chicken serum were measured by flow cytometry as described.^9^ Strains 519/Δ*gtrC* ^III^ or 519/Pgtr^III^C-34A were used as the bacterial targets. The goat anti-chicken IgG (H + L) secondary antibody Alexa Fluor™ 488 (Thermo Fisher) was used as the secondary antibody. The tests were carried out on CytoFLEX S flow cytometer, and the data were analyzed using FlowJo (Tree Star). After gating on bacterial particles, log(median fluorescence intensities, MFI) values were plotted against a lavage dilution factor for each sample, and four-parameter logistic curves were fitted using Prism (GraphPad). Titers were calculated from these curves as the dilution factor giving an above-background signal.

### Statistical analysis

The data are presented as the mean ± standard error of the mean (SEM). Student's *t*-test was used to determine statistical significance between two groups. One-way analysis of variance (ANOVA) followed by Tukey's post-hoc test was used for comparisons among multiple groups. Two-way analysis of variance (ANOVA) followed by Tukey's post-hoc test was used to analyze the effect of two independent categorical factors on a continuous dependent variable. Levene's test was performed to evaluate differences in data dispersion (variance) between the two groups. All the statistical analyses were performed using GraphPad Prism 8 software. *p*-values < 0.05, <0.01, <0.001, and <0.0001 were considered statistically significant and are denoted by *, **, ***, and ****, respectively.

## Supplementary Material

Supplementary_clean.docx Supplementary_clean.docx

## Data Availability

All the data needed to evaluate the conclusions in the paper are presented in the paper and/or the Supplementary materials.
