# Supplementary material for: A single nucleotide polymorphism collapses phase variation and drives short-sighted evolution of O-antigen in Salmonella
Source: Gut Microbes. 2026 Aug 24;18(1):2721178. doi: 10.1080/19490976.2026.2721178 (PMC13505456; doi:10.1080/19490976.2026.2721178)
Supplement: Supplementary file 1 [file KGMI_A_2721178_SM9420.docx]

Table S1 Bacterial strains used in this study

| Strains | Description | Source or reference |
| --- | --- | --- |
| CVCC519 | *S*. Pullorum standard type, isolated from China | Purchased from the China Institute of Veterinary Drug Control |
| CVCC529 | *S*. Pullorum variant type, isolated from the USA |  |
| CVCC530 | *S*. Pullorum variant type, isolated from the USA |  |
| CVCC531 | *S*. Pullorum variant type, isolated from UK |  |
| CVCC79201 | *S*. Pullorum standard type, isolated from China | Provided by the Poultry Institute, Chinese Academy of Agricultural Sciences |
| CVCC533 | *S*. Pullorum standard type, isolated from China |  |
| CVCC534 | *S*. Pullorum variant type, isolated from the USA |  |
| CVCC535 | *S*. Pullorum variant type, isolated from the USA |  |
| CVCC540 | *S*. Pullorum variant type, isolated from French |  |
| *E. coli* DH5α | Host for cloning vector | Takara |
| *E. coli* BL21(DE3) | Host for expression vector | Vazyme |
| 530/Δ*gtrC*^Ⅰ^ | *gtrC*^Ⅰ^ gene deletion in CVCC530 | This study |
| 530/Δ*gtrC*^Ⅲ^ | *gtrC*^Ⅲ^ gene deletion in CVCC530 | This study |
| 519/Δ*gtrC*^Ⅲ^ | *gtrC*^Ⅲ^ gene deletion in CVCC519 | This study |
| Re-530/Δ*gtrC*^Ⅲ^ | *gtrC*^Ⅲ^ complementation in 530/Δ*gtrC*^Ⅲ^ | This study |
| 519/OE-*gtrC*^Ⅲ^ | *gtrC*^Ⅲ^ overexpression in CVCC519 | This study |
| 533/OE-*gtrC*^Ⅲ^ | *gtrC*^Ⅲ^ overexpression in CVCC533 | This study |
| 519/Δ*oxyR* | *oxyR* gene deletion in CVCC530 | This study |
| 519/Δ*dam* | *dam* gene deletion in CVCC530 | This study |
| 519GFP | Single-copy chromosomal *gtrABC*^Ⅲ^::*gfp* translational fusions in CVCC519 | This study |
| 519GFP/Δ*dam* | Single-copy chromosomal *gtrABC*^Ⅲ^::*gfp* translational fusions in 519/Δ*dam* | This study |
| 519/Pgtr^Ⅲ^C-34A | *gtrC*^Ⅲ^ promoter -34 C→A mutation in CVCC519 | This study |
| 519GFP/Pgtr^Ⅲ^C-34A | Single-copy chromosomal *gtrABC*^Ⅲ^::*gfp* translational fusions in 519/Pgtr^Ⅲ^C-34A | This study |

Table S2. Primers used in this study.

| **Oligonucleotides** | **Sequence (5'-3')** | **Target/Description** |
| --- | --- | --- |
| **Primers for constructing gene deletion and complemented strains** | |  |
| R-gtrC1-F | TACACGAAACTTCGGGAATGATGCAAGATGCACAACCATTTTATTAAT CTTTTTTTAAAATTGAGGTAATTTAAGTGTGTAGGCTGGAGCTGCTTCG | deletion of *gtrC*^Ⅰ^ |
| R-gtrC1-R | TCACCGTTAATATAGTGGCAGTTACACAGTATGACTGGCAGGTTCTATAT TGGATTTATATGTAATAGTAACAGCCTATCATATGAATATCCTCCTTAG |  |
| R-gtrC3-F | AATATATTGGTAGAATTTACATAGAAACCAAAAAAAGACCTAAGTACAT AATTAAGAATGAGAAGAAAAATGGTTAATGTGTAGGCTGGAGCTGCTTCG | deletion of *gtrC*^Ⅲ^ |
| R-gtrC3-R | CATTTGTGACATACGATATGTAGGAATCCCCGCCGCCCGTTACCCATTGG TGGCGGGGAACATTAATTATACATGAATGCATATGAATATCCTCCTTAG |  |
| R-oxyR-F | AGAAAGGTTGATAGGGATAATCGTTCGTTGCTATGCTACCTATCGCCATG AACTATCGTGGCGACGGAGGATGAATATGTGTAGGCTGGAGCTGCTTCG | deletion of *oxyR* |
| R- oxyR-R | AACACCACCTTTAACTACCCAACCATGGCGGAAGCTTATCGGGTTGCGG CGTTGAACGGCTTAAACCGCCTGTTTTAAGCATATGAATATCCTCCTTAG |  |
| R-dam-F | ACAGCCTAAAGCGCAGGATGCTGTCGGAGCTTTCTCCACAGCCGGAGA AGGTGTAATTAGTTAGTCAGCTGTGTAGGCTGGAGCTGCTTCG | deletion of *dam* |
| R-dam-R | ACCCAGGCGAGCAAAATCAGCCGACAGAATTGAGGGGGCAATCAAATA CTGTTTCATCCGCTTCTCCTTGAGAACATATGAATATCCTCCTTAG |  |
| Re-*gtrC*3-F | CCGGGATCCATGAGAAGAAAAATGGTTAACA | *gtrC*^3^ complementation |
| Re-gtrC3-R | CGCGAATTCTTATTTAATTATTTCCGTAATAT |  |
| **Screening for gene deletions** | |  |
| S-gtrC1-F | CACGAAACTTCGGGAATG | *gtrC*^Ⅰ^ |
| S-gtrC1-R | GACTGGTCGCTGGCAAT |  |
| S-gtrC3-F | GTTCTGTCATGGGTGGG | *gtrC*^Ⅲ^ |
| S-gtrC3-R | GCGTATCCTTGCGTGTA |  |
| S-oxyR-F | CCTGAACGGTTGGGCGAAGA | *oxyR* |
| S-oxyR-R | CCGGGTGAAAGGTAATGATGCTC |  |
| S-dam-F | CCTGAACGGTTGGGCGAAGA | *dam* |
| S-dam-R | CCGGGTGAAAGGTAATGATGCTC |  |
| **Primers for single-base substitution of the gtr^Ⅲ^ promoter** | |  |
| *gtrA*-Up-1F | ggagagctcgatatcgcatgcGCTATCACTAGCCAGCAAAATAATCT | *gtrA* upstream homology arm amplified from template CVCC519 |
| *gtrA*-Up-1R | tcatTGCTAAGTAGTTTGGGACAAAAACG |  |
| *gtrA*-Down-1F | cccaaactacttagcaATGAAAATCTCGTTAGTCGTTCCG | *gtrA* downstream homology arm amplified from template CVCC519 |
| *gtrA*-Down-1R | tcttctagaggtaccgcatgcGGCGTATTCAACTATATCAGTCTTGC |  |
| *gtrA*-Up-2F | ggagagctcgatatcgcatgcGCTATCACTAGCCAGCAAAATAATCT | *gtrA* upstream homology arm amplified from template CVCC530 |
| *gtrA*-Up-2R | ggggctactgatTGCTAAGTAGTTTGGGACAAAAACG |  |
| *gtrA*-F | acttagcaATCAGTAGCCCCAATTGATCGG | *gtrA* |
| *gtrA*-R | catTTCGCATCCCTAAAGACAATGA |  |
| *gtrA*-Down-2F | gtctttagggatgcgaaATGAAAATCTCGTTAGTCGTTCCG | *gtrA* downstream homology arm amplified from template CVCC530 |
| *gtrA*-Down-2R | tcttctagaggtaccgcatgcGGCGTATTCAACTATATCAGTCTTGC |  |
| **Primers for constructing the gtrC^Ⅲ^ gene overexpression plasmid** | |  |
| I-gtrC-F | agaaagaggagaaatactagATGAGAAGAAAAATGGTTAACAATAGATT | *gtrC*^Ⅲ^ |
| I-gtrC-R | ctcagctaattaagcttttaTTATTTAATTATTTCCGTAATATTCTCATTTG |  |
| I-pCDF-F | TAAAAGCTTAATTAGCTGAGCTTGGA | pCDF-J23110 |
| I-pCDF-R | CTAGTATTTCTCCTCTTTCTCTAGTGCTAGT |  |
| **Primers for constructing the GFP reporter strain** | |  |
| GFP-up-F | ggagagctcgatatcgcatgTATGATTCTTTCGATTTCCTTTGTTG | Downstream of *gtrC*^Ⅲ^ |
| GFP-up-R | ctggatcctcccatTGAATGTTATTTAATTATTTCCGTAATATTC |  |
| GFP-F | cattcaATGGGAGGATCCAGCAAAGG | *gfp* |
| GFP-R | caTTATTTGTAGAGCTCATCCATGCC |  |
| GFP-down-F | gatgagctctacaaataaTGTATAATTAATGTTCCCCGCCA | downstream of *gtrC*^Ⅲ^ |
| GFP-down-R | agcttcttctagaggtaccgCGCCACACGGCCATCAGA |  |
| pRE-F | CGGTACCTCTAGAAGAAGCTTGGG | pRE112 |
| pRE-R | CATGCGATATCGAGCTCTCCC |  |
| **Primers for constructing the gtr promoter reporter plasmid** | |  |
| gtr195-F | ttctggaattctaaagatctTGCAAAGCTTTGTGTGTCCCG | Forward primer for amplifying the P_Std_-1/ P_Var_-1 fragment |
| gtr118S-F | ttctggaattctaaagatctAATTAATAAGATAACAATAACTTTAA**A**CTATTGAAT | Forward primer for amplifying the P_Std_-3 fragment |
| gtr118V-F | ttctggaattctaaagatctAATTAATAAGATAACAATAACTTTAA**G**CTATTGAAT | Forward primer for amplifying the P_Var_-3 fragment |
| gtr91-F | ttctggaattctaaagatctCTATTGAATACCACATTATTGATCGTTT | Forward primer for amplifying the P_Std_-4/P_Var_-4 fragment |
| gtr-R | atttctcctctttctctagtTATGAAAATCAGCGGATTCGG | Reverse primer for amplifying the P_Std_/P_Var_ fragment |
| Lux-F | ACTAGAGAAAGAGGAGAAATACTAGATGGG | Amplification of the Lux reporter plasmid |
| Lux-R | AGATCTTTAGAATTCCAGAAATCATCC |  |
| **qPCR** | |  |
| q-*gapA*-F | GTTCTGACGGGTCCATCTAAAG | Detection of *gapA* expression level |
| q-*gapA*-R | GATGTCCTGGCCTTCGTATTT |  |
| q-*gtrC*3-F | CTGATTGGCGTATTCGGATCT | Detection of *gtrC*^Ⅲ^ expression level |
| q-*gtrC*3-R | GTATTGTAGACGCACGGGATAG |  |
| q-*oxyR*-F | ATTCTGGCGCTGGTGAAA | Detection of *oxyR* expression level |
| q-*oxyR*-R | CCCACGGATGATCCTCATAAAT |  |
| **Construction of OxyR expression vector** | |  |
| OxyR-F | cagcaaatgggtcgcggatccATGAATATTCGTGATCTTGAATATCTGG | Amplification of the *oxyR* fragment |
| OxyR-R | gtggtggtggtggtgctcgagAACCGCCTGTTTTAACGCC |  |
| 199-F | GAAGATGGCCACTCTCTGCGCGATCAG | Mutation at amino acid 199 of OxyR |
| 199-R | CTGATCGCGCAGAGAGTGGCCATCTTC |  |
| **Primers for amplifying fragments for EMSA and BLI assays** | |  |
| E1-F | ACTTGCAAGCCGATGCAAAG | Amplification of the biotinylated P_Std_-1/P_Var_-1 fragment |
| E1-R | Biotin-TGCGTATGCATCCCATACACA |  |
| E2-F | Biotin-ACCCACGGCTTAGATGTTCCT | Amplification of the biotinylated P_Std_-2/P_Var_-2 fragment |
| E2-R | AATAATGTGGTATTCAATAG |  |
| E3-F | AATTAATAAGATAACAATAACTTTAAAC | Amplification of the biotinylated P_Std_-3/P_Var_-3 fragment with E1R |
| E4-F | CTATTGAATACCACATTATTGATCG | Amplification of the biotinylated P_Std_-4/P_Var_-4 fragment with E1R |

Table S3. Main constituent of sugar residues in O antigen by GC-MS analysis (area %).

| Linkage | CVCC519 | 519/OE-*gtrC*^Ⅲ^ | CVCC530 | 530/Δ*gtrC*^Ⅲ^ |
| --- | --- | --- | --- | --- |
| t-Tyv | 14.54 | 12.99 | 7.99 | 7.66 |
| 4-Rha(p) | 21.31 | 22.38 | 21.96 | 22.92 |
| t-Glc(p) | 3.23 | 20.51 | 21.78 | 0.17 |
| t-Gal(p) | 6.88 | 3.91 | 2.65 | 4.67 |
| 3-Gal(p) | 23.67 | 9.51 | 9.02 | 26.43 |
| 2,3-Man(p) | 22.81 | 24.98 | 28.38 | 26.56 |
| 3,6-Glc(p) | 4.50 | 2.86 | 2.28 | 4.15 |

Fig.S1 Growth curves of CVCC519 and its derived strains.

Fig. S2 The evaluation of antibody titers infected with live CVCC519 and 519/Pgtr^Ⅲ^C-34A. In each group, the sera were collected from 12 chickens at 30 dpi, the antibody levels (IgG) were detected using a *Salmonella* Group D Antibody test kit. Statistical significance of the antibody levels between two groups was determined by Student’s t-test (ns, not significant).
